# Vitamin D status in medical staff in a German university hospital in comparison to wasteworkers in Northern and its association to quality of life: a prospective four-arm cohort study

**DOI:** 10.1186/s12889-025-24849-9

**Published:** 2025-11-07

**Authors:** Navid Tabriz, Lilo Stroink, Beate Bartner, Dirk Weyhe, Verena Uslar

**Affiliations:** 1 University Hospital for Visceral Surgery, Carl von Ossietzky University Oldenburg, Oldenburg, Germany; 2 Pius Hospital Oldenburg, Oldenburg, Germany

**Keywords:** 25-(OH)D, Quality of life, Risk factors for vitamin d deficiency, Medical staff, Vitamin d supplementation

## Abstract

**Background:**

Vitamin D (25(OH) D) deficiency has a high prevalence in adults in Germany. Due to workplace conditions, hospital staff might be particularly at high risk. Therefore, we determined the 25(OH) D status of medical staff in regard to their field of activity compared to a group of outdoor workers with a focus on quality of life (QoL) and 25(OH)D associated risk factors.

**Methods:**

25(OH) D was measured in February (winter) and September (late summer) 2021 in medical staff of a university hospital in northern Germany (inpatient vs. surgical vs. administrative workers), and in a group of outdoor waste collectors. QoL (SF-36 questionnaire), and potential factors influencing 25(OH)D-status were collected.

**Results:**

For *n* = 108 participants 25(OH)D was obtained at both time points. In February, mean 25(OH)D was 40.02nmo/l (SD 21.78). In September, mean 25(OH) D increased significantly to 68.25 nmol (SD 20.75). A significant positive association between 25(OH) D and age (*p* = 0.036), and with work experience (*p* = 0.036) was detected in February. In September, food supplement substitution and previous traveling were associated with significantly higher serum 25-(OH)D level. There was no difference between the fields of activity, nor an association between 25(OH)D status and QoL.

**Conclusion:**

Despite the expertise of medical staff, data seems to indicate an inadequate 25(OH)D supply among most medical staff and even outdoor workers, which is significantly more pronounced in winter. In addition to healthy sun seeking and eating habits, occupational health physician coordinated educational work and/or Vitamin D supplementation could be adequate interventions to optimize vitamin D supply.

## Background

 Vitamin D (25-hyroxy-vitamin D [25-(OH)D]) deficiency and its consequences especially with regard to bone metabolism is a worldwide issue and is reaching pandemic proportions in Western culture. About 70% of the Canadians, 40% of the Americans and 30% of the Europeans demonstrate vitamin D insufficiency [1–3]. In Germany, almost two-thirds of adults were vitamin D deficient in 2011 [4].

The endogenous Vitamin D synthesis accounts for up to 90% of the total body vitamin D level and it is highly dependent on sunlight (ultraviolet B radiation) and therefore on season and latitude [4]. Thus, for international comparison of vitamin D status the seasonal time of vitamin D determination and the localization of the country must always be taken into account. Furthermore, general sun exposure should also always be considered if vitamin D supply is regarded. It has been shown that shift and indoor workers are at high risk to develop vitamin D deficiency. Thus, medical staff in particular appears to be at greater risk of vitamin D deficiency due to their workplace, work location, and work schedules [5]. But a dedicated assessment of the vitamin D status depending on workplace, especially for medical staff in Northern Europe, has not yet been sufficiently investigated. National surveys provide an estimate of the nationwide prevalence of hypovitaminosis as well as sub analyses with respect to age, sex, or socioeconomic status, but do not provide separate risk assessments of different occupational groups, such as medical staff [4, 6, 7]. Untreated Vitamin D deficiency can lead to reduced quality of life and increased costs caused by associated diseases like autoimmune disorders or frailty, especially in patients with hypovitaminosis [8, 9] and its supplementation may have a small to moderate effect on quality of life when used on a short-term basis in diseased populations. However, the evidence for a beneficial effect of long-term vitamin D supplementation on health-related quality of life is still lacking and a general indication for supplementation is still under discussion [10]. Nevertheless, a targeted use may be reasonable in individual indications in the presence of a vitamin D3 deficiency (LANGE).

The primary aim of our prospective longitudinal study was to determine the vitamin D status of employees in a university hospital situated in Northern Germany dependent on their field of activity at different seasonal time points in comparison to a group of permanent outworkers. The secondary aim was to investigate association between vitamin D status and quality of life and the impact of diet and leisure activity as depending variables on Vitamin D status.

## Methods

### Study design

The prospective study was conducted as a four-arm cohort study with two survey dates in winter, February, and late summer/early autumn, September 2021, at a hospital in northern Germany. Three fields of activity in the hospital and one non-clinical occupational group with external workplace (WA) (employees of the local municipal waste collection company) were compared. The clinical staff worked at the Pius Hospital Oldenburg, University of Oldenburg, and were distributed between the areas of inpatient care (IC), surgical care (OR) and administrative activities (AD). The inpatient staff was composed of the occupational groups of nursing, physiotherapy, and physicians with exclusively inpatient activities. Operating room nurses, anesthesiologists, and surgeons made up the OR group. The AD group consisted of registration, organization and Information Technology employees. We chose the groups with the following reasoning in mind: The AD group works normal hours in offices with windows mostly in sitting positions. In comparison, the IC group should have the same amount of exposition to daylight when working in the day shift. However, due to shift work, they are not always exposed to sunlight and also cover weekend shifts. Also, they might be more active during their working hours than the AD group. The OR group also works in shifts and weekend services, and during working hours they mostly stay in the OR with little to no exposure to daylight. Lastly, the WA group mostly works outdoors with direct exposure to sunlight. However, in the winter months working hours are often during the early dark morning hours. The use of sun protection in summer was not recorded. The amount of body covering worn by outdoor workers was also not explicitly surveyed. However, in Germany, regulations with regards to protective work wear are very strict. In summer time, t-shirts are possible. It should be mentioned here that they spend almost the entire 8-hour working time outdoors. Our collectives therefore reflect a cross-section of the “normal” workers in a hospital as well as outdoor workers.

Further inclusion criteria were age of 18 years and older, and no vitamin D supplementation. The first vitamin D measurement was performed in February. In case of vitamin D insufficiency (25-(OH) D < 30nmol/L (20 µg/L)) participants were informed for ethical reasons. It was up to them whether they started a vitamin D substitution or not. Before the second measurement time point in summer, the participants were specifically asked whether they were taking vitamin D. In the event of a supplementation, these participants were excluded from further evaluation.

To assess the general quality of life, the short form health 36 (SF-36) questionnaire was used [11]. In addition, to check for potential factors known to influence vitamin D supply, the participants filled out a self-designed questionnaire including information on personal details, occupational factors, sun and leisure behavior, and dietary habits. Personal information included age, gender, weight, height, calculated body mass index (BMI), skin type according to Fitzpatrick [12], and medication intake. The occupation-based information was related to the weekly working hours, shift work, the field of activity, working years, and the daylight exposure at workplace. Leisure time behavior was queried in terms of time and length of outdoor activity, and travel behavior. Dietary behavior was assessed in terms of frequency of consumption of foods containing vitamin D, such as eggs, dairy products, cheese, fish or meat, margarine, orange juice, and consumption of nicotine and food supplements. The mentioned procedure was repeated in September.

### Vitamin D analysis

After giving informed consent, participants underwent venous blood sampling at both data collection time points. The blood samples were frozen until analysis. The blood tubes were stored, transported and processed in a cool place protected from sunlight directly after sampling according to the predefined standards of the laboratory. At the end of each survey time, the blood samples were analyzed collectively. 25(OH)D was analyzed using the Elecsys FS Roche assay. The lower detection limit was 3 µg/L (7.5 nmol/L). Vitamin D levels were assessed according to the classification of Holick [13] which is also accepted by the National Institute of Health (NIH) and the Robert Koch Institute [14, 15]. According to these sources, values below 12 µg/L (30 nmol/L) correspond with a deficient supply, serum levels between 12 and 20 µg/L (30–50 nmol/L) indicate inadequate supply and values above 20 µg/L (50 nmol/L) represent adequate supply, and values above a level of 50 µg/L (125 nmol/L) indicate possible oversupply with potential side effects.

### Statistics

The basis for the power calculation was vitamin D values collected over 18 months from the company physician (mean: 21.8 µg/L; standard deviation: 10.9; variance 118). Based on the data, the calculated effect size was 0.25. According to G*Power (version 3.1.9.4; Heinrich Heine University, Düsseldorf, Germany), a study design with two measurement time points with a total of four groups and a p-value of 0.05 and a power of 0.8 resulted in a total case number of 112 subjects for an ANOVA with repeated measures and analysis of between-subject factors (occupational groups). This corresponded to a group size of approximately 30 participants. In order to have sufficient values for the second survey, even after participants had dropped out, and to be able to map a normal distribution, we planned with 40 subjects per group.

Statistical analysis was performed with the software package SPSS Statistical Software (version 27; SPSS, Chicago, IL, USA).

Descriptive analysis of the study group and the parameters collected was performed, including absolute and relative frequencies, means, minima, maxima, standard deviations (SD), and 95% confidence intervals (CI) as applicable for categorial and scalar data. The vitamin D supply of the different activity groups was compared by means of an ANOVA with repeated measures.

To investigate the association between other variables and 25(OH)D levels, Pearson’s correlation coefficient was calculated for the metric variables (age, body weight, body height, BMI, working years, working time without daylight, distance of duty). In addition, 2-way ANOVAS were calculated for the Overall Score and the Fatigue subscale of the SF-36 questionnaire, respectively, in relation to field of work and status of vitamin D supply (deficient vs., inadequate vs. adequate) for the winter measurement.

To evaluate comparability between groups with regards to participant characteristics, either a one-way ANOVA (for metric variables age and BMI) or the Chi^2^-test (for categorical variables gender and Fitzpatrick skin type) were calculated for both time points.

A p-value < 0.05 was judged to be statistically significant. For all variables used in ANOVAS assumptions of normality and variance were tested beforehand using Shapiro-Wilk tests and Levene’s test, respectively. Assumptions for using ANOVAS were met in every case. For multiple testing corrected p-values using the correction according to Benjamini and Hochberg were used [16]. Only the corrected p-values are reported below.

## Results

### Participants

**Fig. 1:**
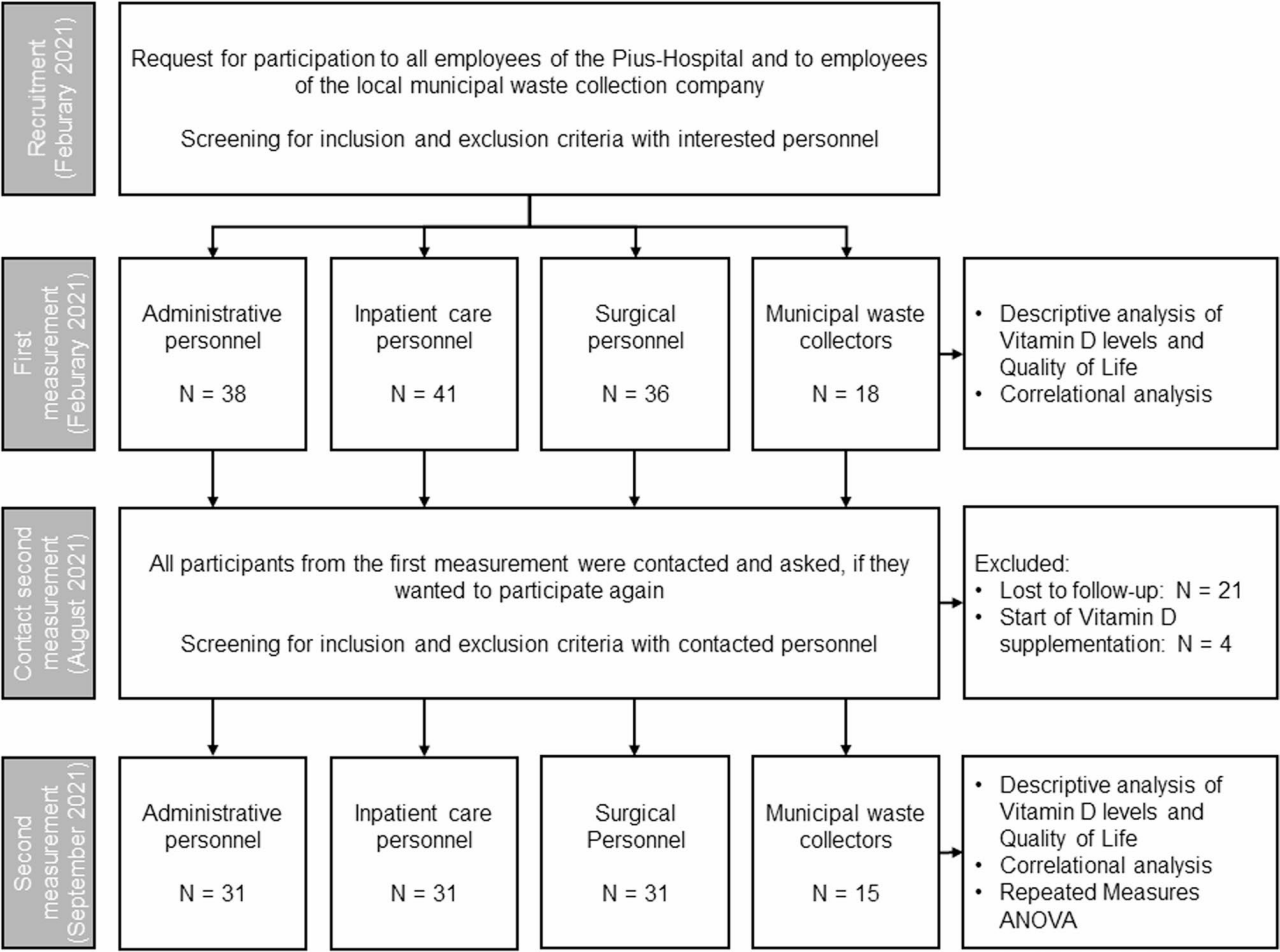
Study flowchart

A total of *n* = 133 participants were included in the first data collection time point in February (winter) 2021 (see also Fig. 1). The majority of participants (*n* = 115) were employed in the clinical field and were distributed between inpatient activities with 41 employees, administrative activities with 38 participants, and operational activities with 36 employees. The proportion of women among the clinical staff was 81%. *N* = 18 participants belonged to the non-clinical occupational group (employees of the local municipal waste collection company). These participants were exclusively male. The skin types in the respective groups were comparable. Overall, 86–94% of all participants had skin types 2 and 3 (fair skin/darker white skin).

In the second survey in September (summer) 2021, 108 participants could be followed-up, corresponding to a response rate of 81.2%. Mostly, loss-to-follow-up was due to the vacation time. The participants were evenly distributed among the clinical work areas with 31 employees in the inpatient area, 31 in the operational and 31 employees in the administrative work area, as well as 15 employees from waste management company. Among the clinical staff, 77.4% of the participants were female. *N* = 4 participants started vitamin D supplementation after the first survey, and where therefore excluded. The characteristics of the different study groups are presented in Tables 1 and 2 for each time points.

**Table 1:** Characteristics of the study groups (02/2021); given are either mean values and the standard deviation (m, (SD)), or the number and percentages (n (%))

	AD* (*n* = 38)	OP* (*n* = 36)	IC* (*n* = 41)	WA* (*n* = 18)	General (*n* = 133)
Age in Years; m, (SD)	42,4 (13,5)	40,1 (12,4)	44,0 (13,1)	48,5 (11,3)	43,1 (12,9)
Female, n (%)	34 (89,5%)	25 (69,4%)	35 (85,4%)	0	94 (70,7%)
Male, n (%)	4 (10,5%)	11 (30,6%)	6 (14,6%)	18 (100%)	39 (29,3%)
BMI in kg/m^2^; m (SD)	25,2 (4,3)	25,3 (4,4)	26,9 (5,3)	28,9 (4,9)	26,2 (4,9)
Fitzpatrick skin type:					
Skin type 1; n (%)	0	1 (2,9%)	4 (9,8%)	0	5 (3,8%)
Skin type2; n (%)	17 (45,9%)	21 (60%)	24 (58,5%)	11 (61,1%)	73 (55,7%)
Skin type 3; n (%)	17 (45,9%)	10 (28,6%)	12 (29,3%)	6 (33,3%)	45 (34,4%)
Skin type 4; n (%)	3 (8,1%)	2 (5,7%)	1 (2,4%)	1 (5,6%)	7 (5,3%)
Skin type 5; n (%)	0	0	0	0	0
Skin type 6; n (%)	0	1 (2,9%)	0	0	1 (0,8%)

*Employees are split with regards to:

*AD* Administrative duties, *IC* Inpatient care duties, *OP* Surgical care duties, *WA* Waste collection duties

**Table 2:** Characteristics of the study groups (09/2021)); given are either mean values and standard deviations (m, (SD)), or the number and percentages (n (%))

	AD (*n* = 31)	OP (*n* = 31)	IC (*n* = 31)	WA (*n* = 15)	General (*n* = 108)
Age in Years; m (SD)	42,4 (14,4)	40,8 (12,3)	43,6 (13,5)	49,1 (11,9)	43,23 (13,3)
Female; n (%)	27 (87,1%)	20 (64,5%)	25 (80,6%)	0	72 (62,6%)
Male; n (%)	4 (12,9%)	11 (35,5%)	6 (19,4%)	15 (100%)	35 (31,3%)
BMI in kg/m^2^; m (SD)	25,4 (4,4)	25,4 (4,5)	26,8 (5,1)	29,0 (5,2)	26,3 (4,9)
Fitzpatrick skin type					
Skin type 1; n (%)	0	1 (3,2%)	3 (9,7%)	0	4 (3,7%)
Skin type 2; n (%)	17 (54,8%)	16 (51,6%)	17 (54,8%)	10 (66,7%)	60 (55,6%)
Skin type 3; n (%)	13 (41,9%)	11 (35,5%)	10 (32,3%)	5 (33,3%)	39 (36,1%)
Skin type 4; n (%)	1 (3,2%)	2 (6,5%)	1 (3,2%)	0	4 (3,7%)
Skin type 5; n (%)	0	0	0	0	0
Skin type 6; n (%)	0	1 (3,2%)	0	0	1 (0,9%)

*Employees are split with regards to:

*AD* Administrative duties, *IC* Inpatient care duties, *OP* Surgical care duties, *WA* Waste collection duties

### Vitamin D analysis

In the analysis of vitamin D level with regard to the field of activity and the time of year, only participants who took part in both surveys were included within the framework of an ANOVA with repeated measures (*n* = 108; see also Fig. 1). The analysis associating factors on vitamin D level and quality of life included the entire test subjects of a respective measurement time point (*n* = 133 and *n* = 108, respectively).

**Fig. 2:**
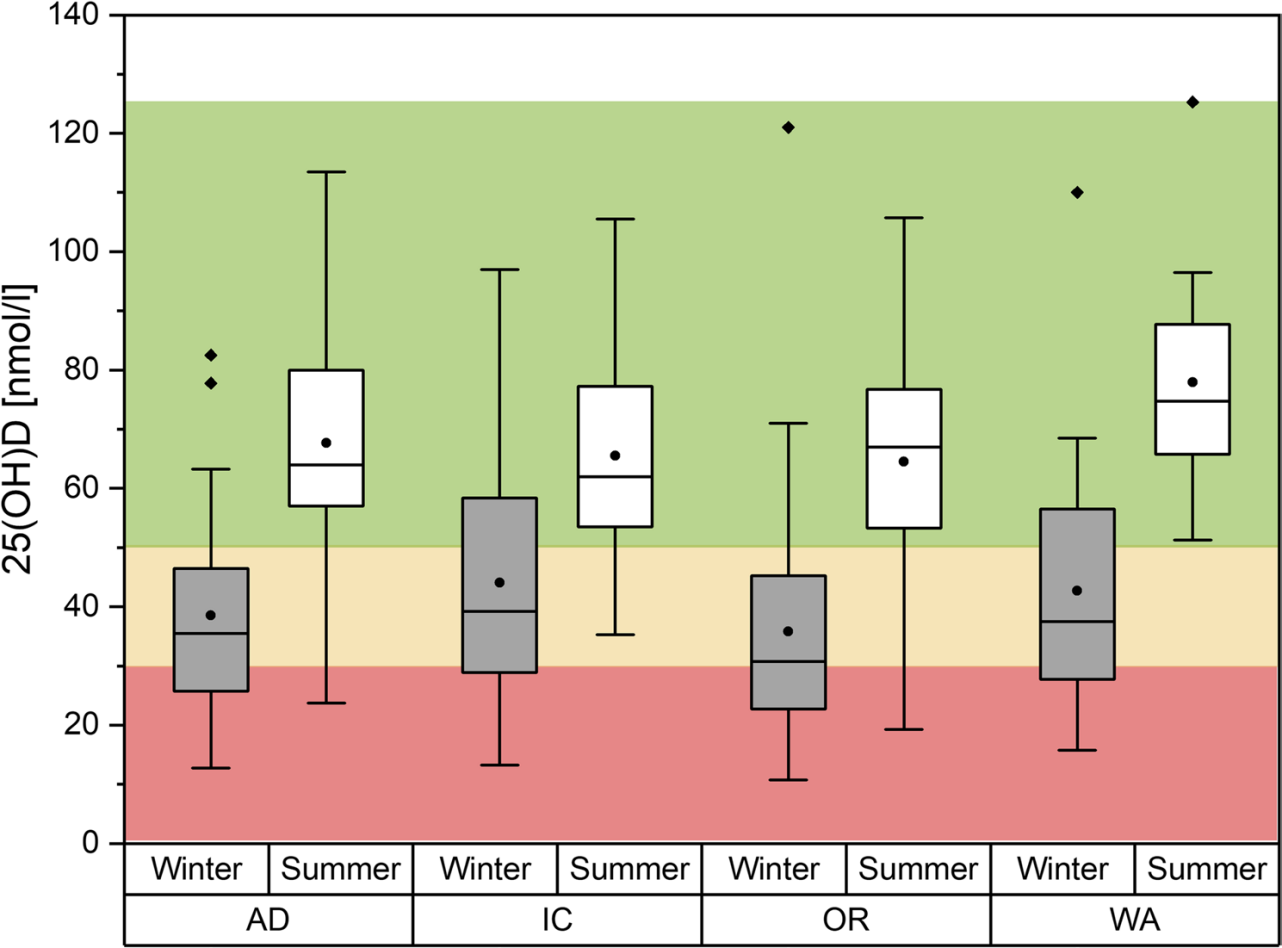
25-(OH)D-Supply (nmol/L) with regard to the field of activity in winter (gray boxes) and summer (white boxes). The boxes indicate the 1.and the 3. quartile as well as the median, the whiskers indicate the 1,5 interquartile range. Means are given as black dots. Statistical outliers are drawn as diamonds. Red, yellow and green background indicate the range for deficient, inadequate, and adequate supply, respectively. AD: administrative duties, IC: inpatient care duties, OP: surgical care duties, WA: waste collection duties

When comparing the investigated activity sectors, the operations room employees (OR staff) had the lowest vitamin D level in winter. The average 25(OH) D concentration was lowest in this group at 34.6 nmol/L (95% CI: 26.8–42.4), and the proportion of employees with severe vitamin deficiency was highest with about 50% being in the range of a deficient supply, and about 25% having inadequate and adequate supply, respectively (Fig. 2). The administrative activity employees (AD group) showed also very low levels of 25-(OH) D, with a mean level 40.9 nmol/L (95% CI: 31.3–50.6) and only about 25% being in the range of adequate supply. IC staff showed the highest mean 25-(OH) D concentration in winter (mean: 43.6 nmol/L; 95% CI: 36.6–50.6), and had the largest proportion of participants with adequate 25-(OH)D levels. Among waste collector employees (WA staff), a mean 25(OH) D level of 41.7 nmol/L (95% CI: 29.2–54.2) was measured. Individual levels in this group ranged from a minimum of 15.8 nmol/L to a maximum of 110 nmol/L. In both latter groups, about 25% of all participants showed deficient supply.

In the summer survey, mean 25-(OH) D levels for the three hospital groups ranged between 64.2 nmol/L and 69.0 nmol/L (see Fig. 2). In all three groups more than 75% of all participants showed adequate 25-(OH) D levels. Mean 25-(OH) D levels for WA staff was 77.9 nmol/L, and all participants were in a range corresponding to adequate supply.

A repeated-measures ANOVA with Greenhouse-Geisser correction showed a significant increase in vitamin D level over the summer months (F (1, 104) = 141.84; *p* < 0.001) in all groups. The repeated-measures ANOVA showed no significant difference in vitamin D level between the different fields of activity (F (3,104) = 0.804; *p* = 0.494).

### Factors associated with the vitamin D level

**Fig. 3:**
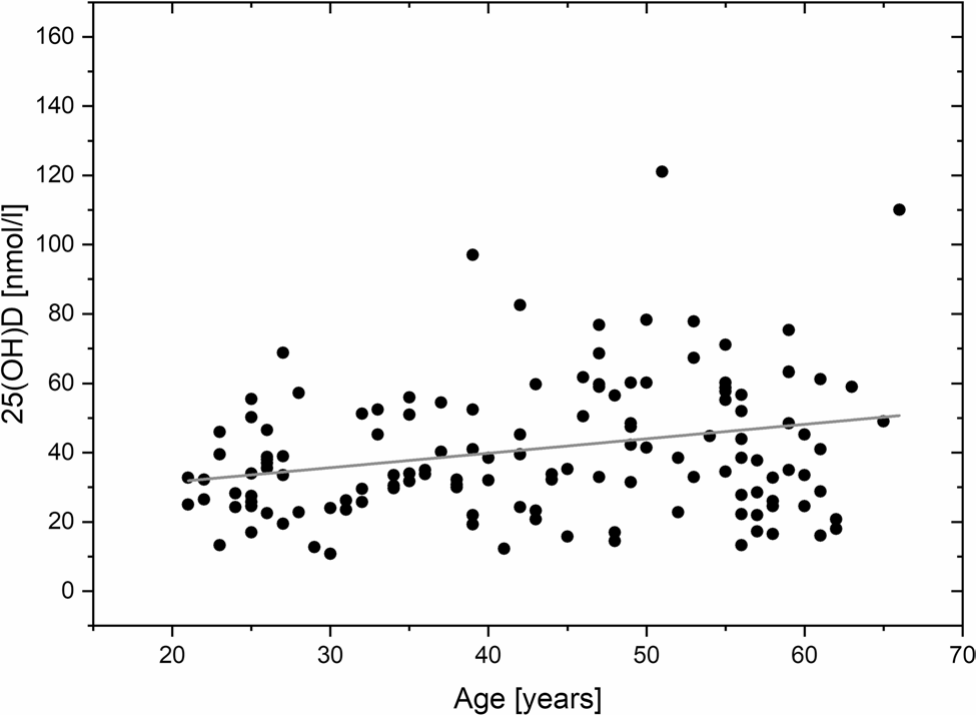
Relation between the age and the 25-(OH) D level in the winter (*n* = 133). The gray line indicates the linear fit

When further variables possibly influencing serum 25(OH) D concentration were examined, a significant association between age and the level of 25(OH) D was found in Pearson’s correlation analysis (r (132) = 0.229; *p* = 0.007; see also Fig. 3) in winter. In addition, there was a significant relationship between work experience in the occupational field and the 25(OH)D level (r (128) = 0.282; *p* = 0.036). Participants of older age and with higher years in the occupational field had higher 25(OH)D concentrations in serum. When tested for correlation, a significant relationship between person’s age and the work experience could be revealed (r (127) = 0.616; *p* < 0.001).

To check for potential confounders on vitamin D status, the intake of food supplements was asked. The participants who reported taking nutritional supplements consisted of: magnesium (5x), vitamin D (4x), BCAA (branch chained amino acid) (2x), OPC (Oligomer Proanthocyanin) (2x), vitamin B12 (2x), β-carotene (1x), iron (1x), silica (1x), multivitamins (1x), omega-3 fatty acids (1x), orthomol-immun (1x), vitamin C (1x), no indication (1x).

**Fig. 4:**
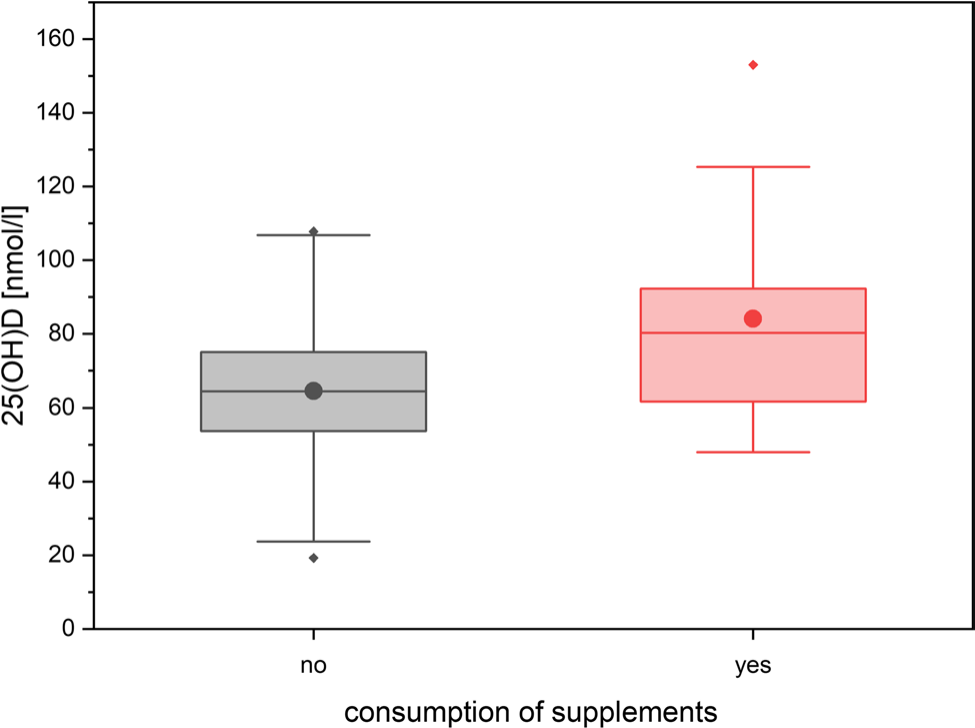
25-(OH)D levels depending on consumption of food supplements in the summer. The boxes indicate the 1. and 3. quartile as well as the median, the whiskers indicate the 1,5 interquartile range. Means are given as black dots. Statistical outliers are drawn as diamonds

At the second survey in summer, there was a significant association between the consumption of food supplements and serum 25-(OH) D level with higher serum concentrations in the group taking food supplements (mean: 81.1 nmol/L; 95% CI [68.4–93.7]) vs. without intake of supplements (mean: 64.6 nmol/L [95% CI: 60.9–68.3]; two sample t-test, t (101) = −3.18; *p* < 0.001) (see Fig. 4).

Since the second survey took place in September after the usual summer vacation, participants were asked about their vacation destination in the last 6 weeks. After excluding participants who started vitamin D substitution therapy (*n* = 4), *n* = 39 out of *n* = 104 participants reported that they had spent their vacation in more sunny regions, i.e., Baltic Sea, North Sea, Mediterranean Sea coast. Participants who reported having traveled in the last 6 weeks showed significantly higher 25-(OH)D levels than the participants who remained home. Among them, the mean 25-(OH) D level was 64.5 nmol/L; 95% CI [59.8–69.2] compared with 72.7 nmol/L; 95% CI [66.9–78.4] in the group that had not traveled.

All other variables on workplace factors such as shift work, leisure time behavior, and dietary habits as well as gender showed no interaction with serum 25(OH)D levels.

### Vitamin D level and quality of life

The results of the SF-36 questionnaire at each time point are depicted in Table 3.

**Table 3:** SF-36 score for the first (02/2021) and second (09/2021) measurement compared to age adjusted references for Germany [17]. Given are mean values and standard deviations (m, (SD)

	02/2021 (winter)	09/2021 (summer)	Reference [18]
Physical functionality; m, (SD)	90,2 (14,2)	92,3 (12,4)	89,5
Physical role function; m, (SD)	91,8 (18,6)	90,3 (23,3)	85,5
Social functionality; m, (SD)	82,8 (24,2)	85,3 (20,2)	85,6
Psychological well-being; m, (SD)	74,5 (14,6)	76,3 (16,0)	72,8
Emotional role function; m (SD)	90,1 (22,4)	91,3 (21,6)	86,8
Vitality; m, (SD)	54,8 (16,4)	56,9 (19,6)	60,7
Pain; m, (SD)	83,6 (21,8)	85,3 (20,1)	75,3
General Health; m, (SD)	70,3 (15,7)	71,7 (17,0)	69,9
Total Score; m, (SD)	79,8 (12,5)	81,1 (12,7)	79,3

The overall score was in summer slightly higher than in winter, (81.1 (SD 12.7) versus 79.8 (SD 12.5)). No significant relationship between 25(OH)D level and health-related quality of life could be demonstrated at any time point. Correlation analysis showed no significant relationship between serum concentration and the score obtained in the total score of the SF-36 and in the subscale fatigue.

**Fig. 5:**
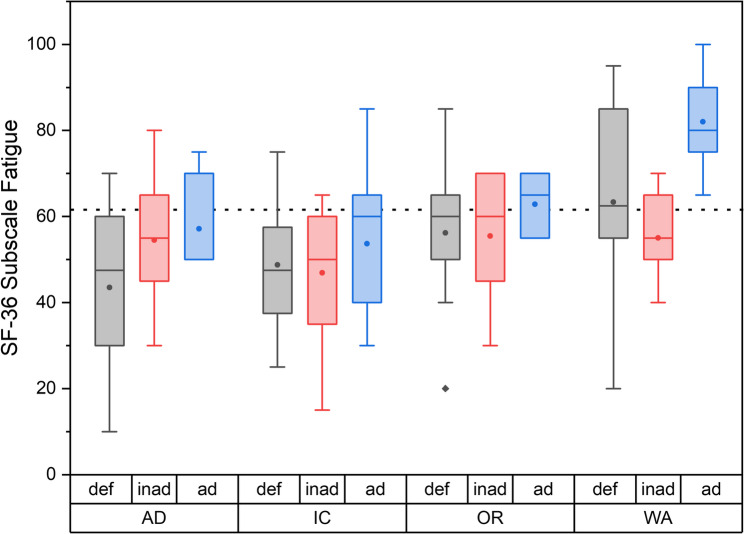
Fatigue subscale of the SF-36 questionnaire in relation to field of work and vitamin D status (deficient = def; inad = inadequate; ad = adequate) for the winter measurement. The boxes indicate the 1.and the 3. quartile as well as the median, the whiskers indicate the 1,5 interquartile range. Means are given as black dots. Statistical outliers are drawn as diamonds. AD: administrative duties, IC: inpatient care duties, OP: surgical care duties, WA: waste collection duties

Adjusted norm values for the age range between 40 and 49 years for Germany [19] were comparable to the mean total score of the participants in our study. Also, in the subscales physical functionality, social functionality, psychological well-being, emotional role function and general health, the score ranks were comparable between the German norm values and the collected questionnaires. Notably, scores for fatigue were lower than in the reference population, in winter as well as in summer. This is mostly due to low scores in the AD and the IC groups in winter (see also Fig. 5). These differences in fatigue values were significant in a two-way ANOVA between vitamin D status (deficient, inadequate or adequate; *p* = 0.005), and field of work (*p* < 0.001). Post-hoc tukey test showed a significant difference between WA and AD workers (*p* = 0.017), and WA and IC groups (*p* = 0.004).

## Discussion

Vitamin D deficiency is a widespread issue with potential effects on quality of life. This study highlights the high rate of vitamin D deficiency in Northern Germany in general and for medical staff as a special risk group especially in wintertime. Out of all participants, only 25% had Vitamin D levels in normal range in winter versus 80% in summer. Our observations, though pertaining only to a relatively small collective, are roughly in line with nationwide studies describing a critical vitamin D supply in large parts of the German population. For Northern Germany, where Oldenburg is located, Diehl et al. evaluated more than 99,000 blood samples and observed that men and women of all age groups were affected by a suboptimal supply, which was particularly pronounced in the months with little sunshine. In winter, the proportion of patients with a severe vitamin D deficit reached 30% [20]. Also, the RKI (Robert Koch Institute) surveyed the nationwide vitamin D supply between 2008 and 2011 in Germany. They reported a high prevalence of vitamin D deficiency, which was strongly dependent on the seasonal time. 8.3% of the population was deficient and 65.8% had an optimal supply in the summer months. In the winter months, these proportions shifted to 52% deficiently and 17.6% optimally supplied. Higher BMI, lack of sport or higher media use were associated with an increased likelihood of Vitamin D deficiency [4].

Several studies from different countries have investigated vitamin D status in medical employees. Critical vitamin D supply was found among physicians in all studies. Vitamin D deficiency in medical staff (< 20 µg/L/50nmol/L) was measured in 25% in Boston [21], 47% in Pakistan [22], and 77% in Israel [23]. In a recent systematic review, Sowah et al. highlighted a higher rate of vitamin D deficiency and insufficiency in medical staff, which was even higher in medical residents and health care students [5]. We could also show that the younger participants seem to be more likely to be affected by vitamin D insufficiency and that vitamin D level seems to increase with age and work experience. This is quite surprising as the vitamin D synthesis capacity of the skin physiologically declines with age [24, 25]. Epidemiological studies indicate a higher prevalence of hypovitaminosis in the elderly, which is evident in women aged 65 years and older [4, 6]. One possible explanation for our findings could be that the age range in this study group was between 21 and 66 years and therefore too young to reflect the biological aging process and epidemiologic trends. Another explanation could be that with increasing age and experience the individual care and health sensitivity to potential hypovitaminosis increases. In this study, especially employees working in the operation theater seem to be at some risk for vitamin D deficiency, which is not surprising as this study group had the lowest sun exposition and a high rate of shift work, which are known factors for vitamin D deficiency [5].

For international comparisons of vitamin D supply different geographical location and different climatic zones, especially with regard to sunshine duration, solar zenith angle as well as divergent working conditions and dietary habits should always be kept in mind. The strength of our study is the longitudinal measurement of vitamin D at clinically significant time points. It is well known that the lowest vitamin D level are measured at the end of winter and spring and the highest in late summer and the beginning of autumn [26, 27] but most of the studies dealing with vitamin D supply do not report the seasonal time in which serum vitamin D was assessed, or store the blood samples for a long amount of time [5]. Therefore, we can state that the time point of vitamin D determination was representative for a longitudinal seasonal measurement.

Darker skin types especially African Americans and Asians have the lowest vitamin D levels but for ethnic comparisons and associations, the geographical location, genetic and cultural factors must also be considered [28, 29]. In contrast, a vitamin D survey from the United Kingdom including 1414 Caucasian females showed that light skin types 1 and 2 have significantly lower levels auf 25-(OH)D compared to darker skin types 3 and 4 [30]. In our respective groups, over 85% of the participants had skin types 2 and 3. However, there was no difference in the distribution of skin types between the four groups. Thus, we can conclude that the skin type played a subordinate role as a confounder.

Especially in the winter months, UV radiation is negligible for locations at high latitude (>35° N) [31]. The geographical localization of the place of investigation, Oldenburg, is placed at 53°N. This fact is also reflected in our study, as vitamin D deficiency was also found in the outdoor working group in winter. On the other hand, this study group had a 25(OH)D level in optimal range in summer. In addition, it was found that those who had spent their vacations in warmer sunnier regions in summer seem to have a better supply of vitamin D., This highlights the important role of sunlight in the physiology of vitamin D. Nutritional supplementation seems to have a general positive impact on serum vitamin D level even if patients who have taken vitamin D supplements were excluded from the analysis. It seems that people who take food supplements generally tend to lead a healthier lifestyle [32] and possibly pay attention to healthy sun exposure.

Vitamin D is involved in a variety of mental and physical processes, some of which have a significant impact on quality of life [19]. This relationship has mostly been studied in the context of individual chronical diseases. Clinical studies report a significant association between low serum vitamin D levels and subjectively worse perceived health-related quality of life. This association was demonstrated in patients requiring dialysis as well as in patients with inflammatory bowel disease or rheumatoid arthritis and was independent of disease severity. A strong association was found particularly for psycho-emotional aspects of quality of life [33–35]. In contrast, other studies found no direct association between vitamin D deficiency and lower quality of life, but suggest that hypovitaminosis occurs as a result of poorer health, which is also the cause of lower vitality, more pain, and poorer role function [36]. Only few studies have examined the relationship between vitamin D supply and quality of life in the healthy general population. The study by Tepper et al. (2016) was one of those few and reported that serum levels of 25-(OH)D were associated with self-rated health using the HRQOL-4 and with physically unhealthy days in healthy high-tech male workers [27]. In contrast, in our study, the participants rated their health-related quality of life measured with the SF-36. No significant association could be found between vitamin D supply and quality of life. The QoL in our study population was comparable to the age-adjusted values of the SF-36 [19]. Only in the “fatigue” subscale a poorer value compared to the German population was seen, both in summer and in winter. vitamin D deficiency can be a factor in fatigue symptoms. However, despite the involvement of vitamin D in regulating mechanisms governing fatigue, other factors could also play a role and there is also conflicting data regarding the positive effect of vitamin D supplementation on fatigue symptoms [37]. Our data suggests that vitamin D deficiency might play an important role in the development of fatigue, since all participants with adequate vitamin D levels show better fatigue scores in general. However, the fact that we only observe a significant relationship in winter seems to indicate that vitamin D is only one of many factors regulating fatigue.

### Limitations

Since vitamin D synthesis is subject to numerous private, occupational, cultural or geographical influences, the observations of the study cannot be readily transferred to other national or international collectives.

With a number of *n* = 108 participants at both time points, the study group was acceptable with regards to our power calculation, although it was admittedly relatively small. While the clinical groups were comparable in size, the number of participating waste workers was only half the size. Due to the relatively small number of participants, it was not possible to identify weaker associations with vitamin D supply or to perform differentiated sub-analyses. The relatively small study population could also be the reason why the vitamin D level in our study tends to increase with age, which seems to be the opposite of the effects reported in other studies. Also, the waste workers were only male and the hospital workers mostly female. This might have an influence on the outcome, although in prior studies exploration of vitamin D status with regards to gender did not reveal any differences [4]. In Germany, vitamin D enriched food is only permitted for margarines and spreadable fats. Manufacturers of other food groups need permission to fortify them with vitamin D. Certain foods such as milk or mushrooms may be enriched with vitamin D through UV irradiation. It is therefore unlikely that the vitamin D level is significantly influenced by high consumption of vitamin D-enriched food in our collective. Nevertheless, we cannot rule this out with certainty.

We excluded four participants after the second sampling, because they answered that they had started vitamin D supplementation during the test period. Therefore, this should not have an effect on the results of the second sampling period. However, we cannot completely exclude that some participants started vitamin D supplementation, although we specifically asked for this.

We are aware that quality of life is a multi-dimensional construct in which social and economic factors also play a role. However, for ethical reasons, we did not explicitly ask about the social status and economic background of our participants. Nevertheless, our participant collective shows no difference in the social functioning domain of the SF-36 compared to a German reference group. Therefore, at least this factor appears to play a subordinate role.

It is unclear to what extent the results were subject to the influence of the COVID pandemic. Lower values are conceivable in the context of the pandemic due to the restricted opportunities for leisure activities and the increased occupational stress. While the first measurement fell into the period of the second lockdown in Germany, with the ban on long-distance travel, the closure of tanning salons, or a masking requirement in public places, the pandemic situation in Germany eased over the summer. Since the number of hospitalized COVID patients decreased, many bans were lifted, allowing more opportunities for leisure activities, social contacts and travel. Occupational stress in the hospital decreased and opportunities for sunlight exposure improved, but a complete return to everyday life did not occur, especially in hospital staff. This might be an explanation for the higher fatigue rates in the SF-36 questionnaire. In a Slovenian survey of vitamin D status and supplementation during the COVID-19 pandemic it could be shown that Vitamin D supplementation is effective for Vitamin D deficiency prevention but only one-fifth of participants took the supplement consistently over the course of the year and compliance was therefore low [38]. But a multimedia campaign with educational work seemed to be an effective awareness program for increasing supplementation of vitamin D [39].

We are aware that the German healthcare system is already struggling economically. However, keeping their (medical) workforce healthy should be a priority to any hospital. Thus, it would be very beneficial to monitor Vitamin D levels of the hospital staff by an occupational physician, especially in the winter months. At the very least, in order to positively influence the vitamin D status in general, a combination of educational work, food fortification and supplementation should be carried out. Although adherence to vitamin D supplementation is low, especially among people with low socio-economic status, food fortification programs are a meaningful and cost-effective measure to counteract the potential negative consequences of vitamin D deficiency [17, 40].

## Conclusions

In summary, this study tried to show the seasonal vitamin D status of medical staff dependent on their working place in a hospital at high latitude. We cannot distinguish between risk levels in different occupational settings. However, medical staff in general seem to be at high risk of vitamin D insufficiency, especially during the winter months. Sunlight and the intake of food supplements in general have a positive effect on vitamin D status. In a homogenous group of participants without any relevant diseases, quality of life in general seems to not be affected by vitamin D deficiency. However, fatigue symptoms were more common in employees with vitamin D deficiency, especially in winter. In order to positively influence the vitality of employees and counteract the long-term consequences of a vitamin D deficiency, educational work would be a sensible measure. The occupational health physician, for example, could advise on dietary and healthy sun exposure recommendations or vitamin D supplementation could be discussed.

## Data Availability

Data is provided within the manuscript. Data will be made available upon request to the corresponding author.

## Abbreviations

25-(OH) D: 25-hydroxyx-vitamin D
WA: Waste collector employees
IC: Inpatient care employees
OR: Operations room employees
AD: Administrative activity employees
SF-36: Short form health 36
BMI: Body mass index
SD: Standard deviation
CI: Confidence interval
BCAA: Branch chained amino acid
OPC: Oligmere proanthocyanidine
RKI: Robert Koch Institute
UV: Ultraviolet
ANÒVA: Analysis of variance
